# OXTR-Related Markers in Clinical Depression: a Longitudinal Case–Control Psychotherapy Study

**DOI:** 10.1007/s12031-021-01930-7

**Published:** 2021-11-25

**Authors:** Iris C. Reiner, Gerald Gimpl, Manfred E. Beutel, Marian J. Bakermans-Kranenburg, Helge Frieling

**Affiliations:** 1 Department of Psychosomatic Medicine and Psychotherapy, University Medical Center, Untere Zahlbacher Str. 8, 55131 Mainz, Germany; 2 Faculty of Social Work, Darmstadt University of Applied Sciences, Darmstadt, Germany; 3 Institute of Biochemistry, Johannes-Gutenberg University Mainz, Mainz, Germany; 4 Clinical Child & Family Studies, Faculty of Behavioral and Movement Sciences, Vrije Universiteit Amsterdam, Amsterdam, The Netherlands; 5 Molecular Neuroscience Laboratory, Department of Psychiatry, Social Psychiatry and Psychotherapy, Hannover Medical School (MHH), Hannover, Germany

**Keywords:** Depression, Oxytocin, OXTR methylation, Psychotherapy

## Abstract

We investigated stability and change of plasma and urinary oxytocin as well as *OXTR* DNA methylation patterns through psychotherapy. Furthermore, we explored the potential impact of inpatient psychotherapy on oxytocin-related biomarkers and vice versa by differentiating patients who remitted from depression versus non-remitters. Blood and urine samples were taken from 85 premenopausal women (aged 19–52), 43 clinically depressed patients from a psychosomatic inpatient unit, and 42 healthy control subjects matched for age and education at two points of time. Serum and urine oxytocin were measured using standard ELISA, and DNA methylation of the *OXTR* gene was assessed using bisulfite sequencing at the time of admission (baseline) and at discharge and from controls at matched time points. Oxytocin plasma levels were not associated with depression and were influenced by neither time in healthy controls nor psychotherapy in patients. Non-remitting depressed patients had significantly lower oxytocin urine levels before and after psychotherapy treatment. We found significantly lower exon 1 *OTXR* methylation in depressed patients over time and these differences were driven by patients remitting due to psychotherapy. A reverse pattern — higher levels of methylation in remitters — was found for exon 2 *OXTR* DNA methylation. Plasma oxytocin, urinary oxytocin, and *OXTR* DNA methylation patterns were intrapersonally relatively stable. *OXTR*-related factors were seemingly unaffected by inpatient psychotherapeutic treatment, but we found significant differences between remitting and non-remitting patients in urinary oxytocin and *OXTR* DNA methylation. If replicated, this suggests that *OXTR*-related markers may predict inpatient treatment outcomes of clinically depressed patients.

**Supplementary Information:**

The online version contains supplementary material available at 10.1007/s12031-021-01930-7.

## Introduction

According to the recent WHO report on depression and mental disorders, worldwide 322 million people suffer from depression, with increasing rates of 18.4% from 2005 to 2015 (WHO 2017). The etiology of depression is likely to be multifactorial and in recent years, biological causes of clinical depression have been studied increasingly. The role of peripheral oxytocin and markers of the oxytocin receptor gene (OXTR) — the gene that guides the oxytocin receptor — on social affiliation, trust, and mood has been studied extensively in humans, (Bakermans-Kranenburg et al. 2014; Feldman et al. 2011)  suggesting that it may also be involved in clinical depression (IsHak et al. 2011).

Current research on the influence of oxytocin, *OXTR*, and *OXTR* DNA methylation on mental health and depression particularly led to mixed results with unanswered questions: Still, it is unknown whether peripheral oxytocin levels in clinically depressed patients differ from those in healthy persons.

Studies on the role of plasma oxytocin in clinical major depression in humans — outside pregnancy and the perinatal period — portray an inconsistent picture: Ozsoy et al. (2009) and Yuen et al. (2014) reported lower plasma oxytocin levels in depressed females. However, others report increased oxytocin levels in clinically depressed humans compared to healthy control subjects (Parker et al. 2010; Cyranowski et al. 2008) and positive correlations between plasma oxytocin and depressive symptoms (Parker et al. 2010) or, respectively, found no significant differences between depressed versus non-depressed subjects (Lien et al. 2017).

Inter-individual variation in peripheral oxytocin levels might also be attributable to variations in DNA methylation of the *OXTR* gene. Although DNA methylation was originally thought to primarily silence gene transcription, emerging evidence shows that it regulates gene transcription on a more fine-grained level and can affect alternative splicing as well (Macartney-Coxson et al. 2020).

In a sample of children with autism, Gregory et al. (2009) found increased methylation in the promoter region of the *OXTR* gene to be linked with a 20% reduction in *OXTR* mRNA. Little is known about the impact of *OXTR* DNA methylation on plasma oxytocin levels. Two studies cautiously indicate sex- and age-specific associations in small subsamples (Dadds et al. 2014; Rubin et al. 2016). However, findings are inconclusive and more research needs to clarify the influence of the *OXTR* gene and its methylation status on plasma oxytocin levels. Additionally, the influence of the *OXTR* gene and its methylation status on urinary oxytocin needs to be clarified.

Our own research on clinical depression and *OXTR* DNA methylation status showed exon-specific methylation patterns interacting with the *OXTR* rs53576 genotype (Reiner et al. 2015): We concluded that exon-specific analyses are needed to understand and possibly explain contradictory results on associations between depression, peripheral oxytocin levels, and *OXTR* genotypes.

Oxytocin and response to psychotherapy treatment is of high relevance in the research of depression. Differential outcomes in the treatment of the disease are poorly understood (Mojtabai 2017; Sackeim 2001) and little is known on both long-term stability and change through psychotherapy of plasma/urinary oxytocin and *OXTR* DNA methylation. Stability or change through psychotherapy or a potential influence on treatment response of *OXTR* DNA methylation in depressed patients has not been examined at all.

In sum, there is remarkable lack of research on associations between peripheral oxytocin levels (plasma and urinary), related (epi-)genetic markers, and clinical depression. This is where the present study comes in: In a sample of clinically depressed women and carefully recruited healthy female control subjects, we investigated stability and change of plasma and urinary oxytocin as well as *OXTR* DNA methylation patterns. Furthermore, we explored the potential impact of inpatient psychotherapy on oxytocin-related biomarkers and vice versa by differentiating patients who remitted from depressive symptoms through psychotherapy versus non-remitters. We examined (a) stability and change of plasma and urinary oxytocin through psychotherapy in remitters and non-remitters, (b) stability and change of *OXTR* DNA methylation patterns through psychotherapy in remitters and non-remitters, and (c) longitudinal associations between *OXTR* DNA methylation and urinary and plasma oxytocin.

## Materials and Methods

### Sample Description

The total sample consisted of premenopausal 85 women aged 19–52 (*M* = 30.1 years, *SD* = 9.0 years). All participants were Caucasian. The depressed sample consisted of 43 patients, who were recruited from the inpatient unit of a Department of Psychosomatic Medicine and Psychotherapy in Mainz, Germany. The control group entailed 42 healthy control subjects who were recruited by flyers and were matched for age and educational level.

### Participants and Procedure

Inclusion criteria for the patient group were the following: diagnosis of depression and/or dysthymia via the Structured Clinical Interview for DSM-IV disorders: SCID-I and SCID-II (Wittchen et al. 1997). In the control group, women who were currently in psychotherapy or had a mental disorder diagnosis (by SCID-I and SCID-II) were excluded from further study participation. Patients with borderline, antisocial, or narcissistic personality disorders as well as patients with bipolar disorder, psychotic disorders, or eating disorders or substance abuse were not eligible. Moreover, menopausal or postmenopausal, pregnant and breastfeeding women, and subjects who suffered from adrenocorticotrophic, gynecological, and neurological diseases were excluded.

Data collection for the patient group was performed within 4 days after admission (T1) and 4 days before discharge (T2). Treatment duration varied between 5 and 12 weeks (*M* = 8.3, *SD* = 1.7). Healthy control participants were matched for age (+ / −3 years) and education (school degree) as well as for time interval between the two assessments. T2 data is not available for two patients who terminated treatment prematurely.

The study was approved by the Ethics Committee of the State Board of Physicians of Rhineland-Palatinate (Germany). All participants provided their written informed consent to participate in this study.

### Treatment

Depressed patients were treated with a multimodal psychotherapy approach in line with the “Mainz Model” (Beutel et al. 2008). Treatment included two psychodynamic individual sessions as well as two group therapy sessions per week. Additionally, body-oriented, art therapy, and other treatment elements (relaxation techniques, psychoeducational groups) complemented the psychosomatic treatment program.

### Depressive Symptoms and Remission from Depression

All study participants filled out self-report depression module of the Patient Health Questionnaire, the PHQ-9 (Löwe et al. 2004) at T1 and T2. The nine items of the PHQ-9 correspond to the DSM criteria of major depression as “0” (not at all) to “3” (nearly every day).

Depressive symptoms in the clinical group were assessed via the structured version of the 17-item Hamilton Depression Rating Scale (HAM-D) (Hamilton 1967; Potts et al. 1990) by trained and experienced psychologists. As suggested by Kyle et al. (2016), we defined a sum score of the HAMD-17 rating at T2 below 8 to indicate remission of depressive symptoms.

### Oxytocin Measurements in Plasma and Urine

Blood samples were collected in EDTA-treated a tube into which aprotinin was added. Tubes were centrifuged at 2250 g at 2 °C for 15 min. Plasma was then frozen at −80 °C until time of assay. The oxytocin levels were determined using a commercial enzyme immunoassay (Enzo Life Sciences, Lörrach). Preliminary measurements following solid-phase extraction on reversed-phase cartridges revealed oxytocin values close to or below the detection level of the immunoassay in most of the samples. Therefore, we used unextracted blood plasma that was diluted in assay buffer (1:4 and 1:8). This allowed us to measure oxytocin values within the linear portion of the standard curve. It is important to note that oxytocin levels determined in unextracted plasma are always several fold higher than those measured in extracted plasma (Szeto et al. 2011). One reason for this is that the extraction process removes significant amounts of oxytocin that are strongly bound to plasma proteins (Szeto et al. 2011; Brandtzaeg et al. 2016).

Urine samples were concentrated fourfold by solvent extraction with ethanol (final 70% v/v). The samples were evaporated by speed-vac and resuspended in the immunoassay buffer. Spike-experiments with oxytocin showed recovery rates > 95%.

All measurements were performed in duplicates and the concentrations were calculated using Sigmaplot 8.0 (Jandel Scientific) according to standard curves.

### Bisulfite Sequencing of* OXTR*

DNA was extracted from blood samples drawn on admission (T1) and on departure (T2) from the inpatient treatment or at similar time points in the healthy control women. All blood samples were drawn in the morning while probands were fasting.

Details on DNA extraction, bisulfite conversion, PCR amplification, and sequencing strategy as well as primer sequences and temperatures and information on the amplified fragments are provided in our previous report on *OXTR* DNA methylation in depressed women (Reiner et al. 2015). In short, DNA was extracted from frozen EDTA whole blood samples using QIAamp® DNA Blood Mini Kit (Qiagen AG, Hilden, Germany) according to the manufacturer’s protocol. 500 ng of genomic DNA were modified by sodium-bisulfite using the EpiTect® Bisulfite Kit (QIAGEN AG, Hilden, Germany). A nested touchdown PCR was performed to amplify a region of the OXTR gene harboring 71 CpG sites within exons 1 and 2, and intron 1. Primer sequences as well as fragment sizes and chromosomal position are listed in supplementary Table 1.

Subsequently, each PCR product was sequenced using a BigDye® Terminator v3.1 Cycle Sequencing Kit (Applied Biosystems, Foster City, CA, USA) according to the manufacturer’s instructions. After dye-terminator removal with Agencourt CleanSEQ System (Beckman Coulter), the products were analyzed on an Applied Biosystems® 3500xL DNA Analyzer (Applied Biosystems). Sequences were analyzed using the ESME software package to determine DNA methylation levels (Lewin et al. 2004).

### Statistical Analysis

If not otherwise indicated, all data are given as mean (SD). Descriptive statistics were performed and deviance from normal distribution was checked by inspection of histograms and KS-test. Group differences were analyzed using unpaired *t*-tests or chi-square tests — first, to detect differences between patients and controls (Table 2) and in a second step, to detect differences between patients remitting from depression and those who did not remit (Table 3). For associations between oxytocin-related variables and clinical variables, Pearson’s correlation coefficients are reported.

### Oxytocin

As data showed no deviation from normal distribution according to Kolmorogov-Smirnov’s test, parametric tests were employed. We used mixed linear modelling to take into account the different times of measurements, as blood samples were collected at baseline and at the end of treatment before and after a biographical interview. In a saturated model, mean oxytocin levels (before/after interview) were set as dependent variable and group and time point were set as fixed factors. In a second model, main effects of possible confounders (age, hormonal contraception, days since last menstruation, antidepressant use, and depressive symptoms (HAM-D)) were included in the model. Marginal means [EMM] were estimated from the model and possible differences in the EMM were analyzed using Bonferroni-corrected pairwise tests. To assess whether “successful psychotherapy” had an impact on the trajectories of oxytocin levels, we repeated the analyses without possible confounders using a group outcome variable with three levels (controls, depressed patients with remitted symptoms at the end of therapy, and patients without remission).

### *OXTR *DNA Methylation

**Fig. 1:**
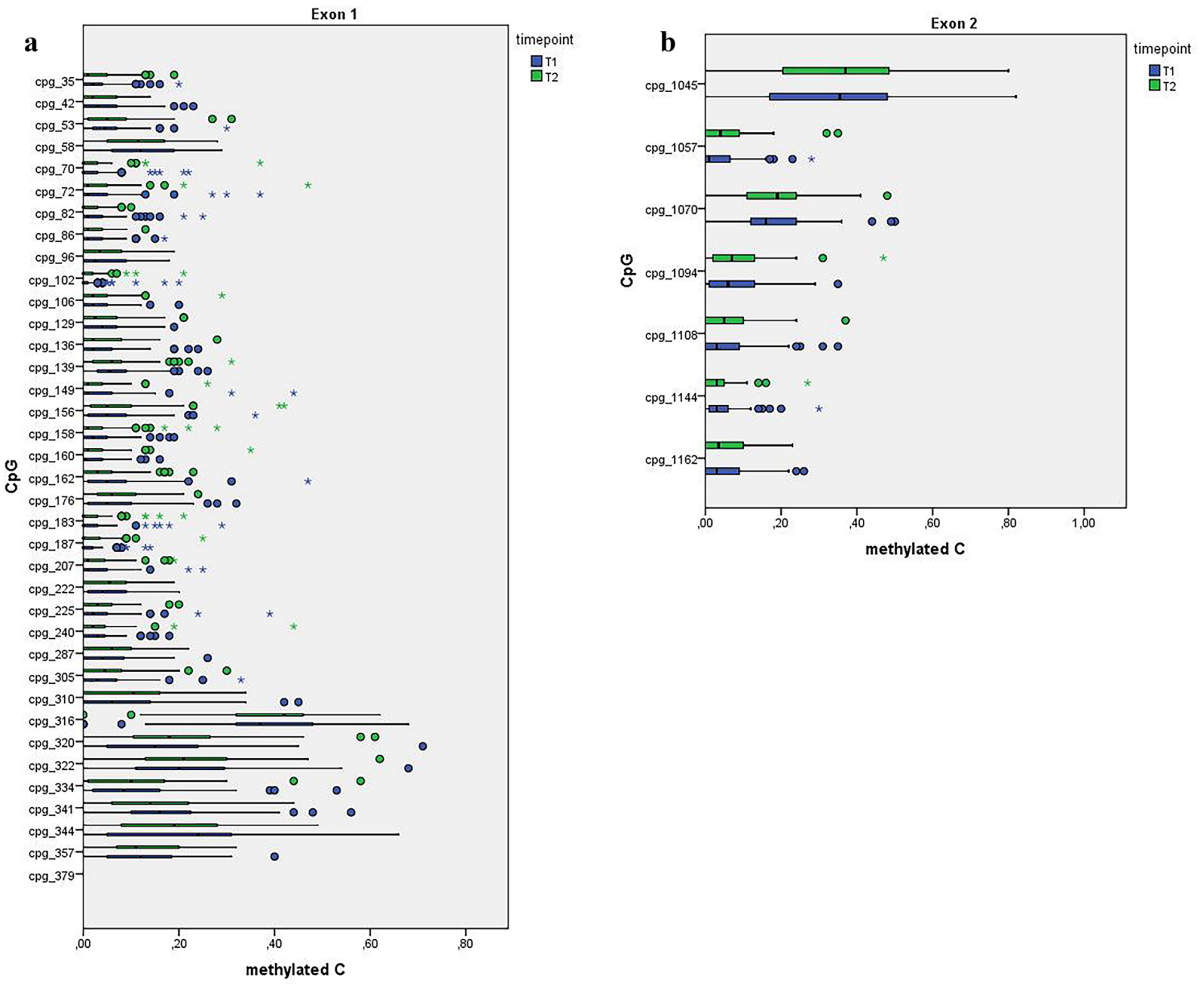
Distribution of methylation across exon 1 and exon 2 of the OXTR gene. Distribution of DNA methylation across OXTR exons 1 (**A**) and 2 (**B**). Fraction of mC is given as beta-value with 1 indicating that all in all copies sequenced, the C at a given CpG position was methylated, while a 0 indicates no methylation in all copies. CpG numbers are related to the first base of exon 1. Methylation values are provided from all study participant (*N* = 84) at T1 (baseline) and T2 (end of study) time points

Bisulfite sequencing yielded methylation values of 69 single CpG sites in the *OXTR* gene. We performed initial quality checks to exclude potentially unreliable measurements: (i) All obtained sequences were screened for sequencing quality using ABI sequence scanner (Applied Biosystems). Samples with a *QV*-value below 20 were measured again. For the final analysis, all sequences were above the *QV* threshold of 20. (ii) Individual CpG sites with more than 10% missing values were excluded from the analysis, leaving 43 CpG sites in the *OXTR* gene — 36 within exon 1 and 7 within exon 2. Intronic CpGs were all excluded due to too many missing values. (iii) No participants had more than 20% missing values; therefore, none of the patients and controls was excluded from the analysis. (iv) Only CpG sites with an inter-individual variance above 0.01 were included into the analysis — this requirement was fulfilled by all 43 remaining CpG sites. A diagram showing the distribution of methylation values over CpG sites is provided in Fig 1.

In order to make our current data comparable to the cross-sectional data already published, we used an analysis approach with mixed linear modelling. Individual CpG sites were computed as repeated measures assuming a scaled identity covariance structure. In models including times of measurement, hierarchical repeated measures (times of measurement within CpG) were computed. F-statistics of fixed effects were used to assess the value of single predictors in the best fitting models.

## Model 1

To analyze the effect of therapy/time on *OXTR* DNA methylation, we used a model containing methylation as dependent variable and CpG, group (depressed vs. control), and time of measurement as well as two-way interactions of all three factors as predictors. This model was repeated for exons 1 and 2 separately. *F*-tests of two-way interactions between time of measurement and group were regarded as the relevant measure for different trajectories of methylation over time.

## Model 2

To test if differences in methylation between the two groups (depressed vs. controls) were still present at T2, we used methylation at T2 as dependent variable and CpG and group as well as their interaction and methylation at T1 as fixed effects. This model was examined for exons 1 and 2 separately.

## Model 3

To test whether differences between controls and patients were depending on therapy outcome, we tested a third model using a three-factorial group variable (controls; patients achieving remission; patients not achieving remission). Using again methylation as dependent variable, we included CpG position, time, and group as well as all two-way interactions of these variables as fixed factors. To test for potential associations between *OXTR* DNA methylation and plasma or urinary oxytocin, we used general linear models also testing for a moderating effect of (a) group or (b) outcome (three-level variable, s. above).

For all analyses, a significance level of *α* = 0.05 was chosen. Analyses were performed using the Statistical Package for the Social Science (SPSS® 22 for Windows, IBM).

## Results

Characteristics of the sample are provided in Tables 1, 2 and 3. Plasma oxytocin levels measured at the four different time points were highly correlated within subjects, while no correlation was found between plasma and urinary oxytocin levels (Tables 4, 5 and 6). No association between plasma or urinary oxytocin levels and clinical variables (age, antidepressant medication, hormonal contraception, or depressive symptoms) (PHQ; HAM-D) (patients only) was found (*data not shown*). PHQ and HAM-D scores were correlated at both time points. Patients receiving antidepressant medication showed slightly higher baseline scores in the HAM-D (antidepressant medication yes vs. no: 16.72 (4.34), *n* = 29 vs. 13.21 (3.70), *n* = 14; *T*_(41)_ = 2.60, *P* = 0.013), but no differences on self-rated depression (PHQ-9) at both time points nor HAM-D scores at T2. Patients receiving hormonal contraception had significantly lower HAM-D as well as PHQ-9 scores at T2, while contraception had no effect on PHQ-9 scores in healthy controls (*data not shown*). To exclude the possibility that changes in the forthcoming analyses were confounded by pharmacotherapy, medication was implemented as covariate/random factor in all models. As not all patients received an antidepressant monotherapy, we repeated all analyses excluding two patients (one treated with the combination of citalopram and mirtazapine, the other with an augmentation of citalopram by lithium). These analyses ended with only negligible differences in the overall results (*data not shown*). Two patients were diagnosed with dysthymia and anxiety disorder. Even though these two patients were comparable with regard to self-rated depressive symptoms, we repeated most analyses without these two patients (*data provided in the supplements*).

**Table 1:** Characteristics of the sample

Baseline characteristics	MDD patients (*n* = 43)		Healthy control women (*N* = 42)		Full sample (*n* = 85)	
	*n*	%	*n*	%	*n*	%
Marital status						
Single	17.0	39.5	11.0	26.2	28.0	32.9
Married/partnered	22.0	51.2	28.0	66.7	50.0	58.8
Divorced/widowed	4.0	9.3	3.0	7.1	7.0	8.2
Cohabitating	28	65.1	31	73.8	59	69.4
Highest educational level						
Middle school	19	44.2	17	40.5	36	42.4
High school/some college	20	46.5	19	45.2	39	45.9
University or postgraduate degree	4	9.3	6	14.3	10	11.8
Employment*						
Unemployed	4	9.3	1	2.4	5	5.9
Employed	23	53.5	29	69.0	52	61.2
Self-employed	1	2.3	2	4.8	3	3.5
Student	12	27.9	10	23.8	22	25.9
Incapable of work	9	20.9	0	0.0	9	10.6
Retired	0	0.0	0	0.0	0	0.0
Hormonal contraception**	7	16.3	19	45.2	26	30.6
Antidepressant medication***	29	67.4	0	0.0	29	34.1
OXTR genotype						
GG	17	39.5	19	46.3	36	42.9
AG/AA	26	60.5	22	53.7	48	57.1

^*^/**/***P < 0.05/ < 0.01/ < 0.001 chi^2^-test

**Table 2:** Comparison between patients and controls (age, days since last menstruation, depressive symptoms)

	MDD patients		Healthy controls		*t(83)*	*P*
	*M*	*SD*	*M*	*SD*		
Age [yrs.]	30	9	30	9	−0.22	0.83
Days since last menstruation						
T1	14	9	13	9	0.26	0.80
T2	16	10	15	9	0.45	0.65
Depressive symptoms (PHQ-9)						
T1	14.84	4.72	2.07	2.06	16.01	< 0.001
T2	10.26	5.01	2.1	1.74	9.99	< 0.001

*PHQ-9*, Patient Health Questionnaire for depressive symptoms. Further details are summarized in the “Results” section

**Table 3:** Comparison between patients remitting vs. non-remitting

	Remitting (*n* = 16)		Non-remitting (*n* = 26)		*t(41)*	*P*
	*M*	*SD*	*M*	*SD*		
Hamilton Depression Scale 17						
T1	14	3.92	16.5	4.6	1.807	0.08
T2	4.5	1.63	12.27	3.46	8.398	< 0.001
Depressive symptoms (PHQ-9)						
T1	14.88	4.59	14.77	4.97	0.069	0.95
T2	6.38	3.05	12.42	4.55	−4.693	< 0.001

*PHQ-9*, Patient Health Questionnaire for depressive symptoms. Remission was defined based on the HAM-D score at the second visit (< 8). Further details are summarized in the “Results” section

**Table 4:** Oxytocin levels in plasma and urine: Means (M) and standard deviations (SD)

							Patients only					
	Healthy controls			Patients			No remission			Remission		
	*M*	*SD*	*n*	*M*	*SD*	*n*	*M*	*SD*	*n*	*M*	*SD*	*n*
Plasma oxytocin T1 before interview	327.05	136.24	41	322.77	122.43	43	319.92	117.22	26	328.06	137.90	16
Plasma oxytocin T1 after interview	345.59	196.14	41	340.26	123.66	43	321.96	113.99	26	367.13	140.26	16
Plasma oxytocin T2 before interview	351.49	127.96	41	354.05	117.29	41	339.24	118.34	25	377.19	115.52	16
Plasma oxytocin T2 after interview	346.00	148.92	41	335.44	115.66	41	320.08	113.47	25	359.44	118.58	16
Oxytocin in urine T1	204.00	127.19	41	156.50	134.58	40	140.46	102.85	24	184.60	178.04	15
Oxytocin in urine T2	228.26	166.48	42	158.59	128.73	41	155.68	147.72	25	163.12	96.15	16

*N* = 84

Oxytocin plasma levels were taken twice at each visit — first sample before and second sample after a biographical interview. Data of patients is given twice, column 2 shows data of all MDD patients, columns 3 and 4 show the data according to the final outcome of the patients. Remission was defined based on the HAM-D score at the second visit (< 8). Differences in *n*’s are due to missing data. All oxytocin levels are given as [pg/ml]. Further details are summarized in the “Results” section

**Table 5:** Intercorrelations between plasma, urinary oxytocin, and depressive symptom variables at time 1

Variable	*n*	*M*	*SD*	1	2	3	4	5	6	7
1.Plasma oxytocin before interview	84	324.86	128.59	—						
2.Plasma oxytocin after interview	84	342.86	162.13	0.82**	—					
3.Oxytocin in urine	81	180.54	132.25	−0.10	−0.17	—				
4.Days since last menstruation	84	13.69	9.24	0.00	0.00	0.02	—			
5.PHQ-9	85	8.53	7.38	0.06	0.01	−0.15	0.07	—		
6.HAM-D	43	15.58	4.43	−0.01	−0.22	0.14	−0.02	0.31*	—	
7.Age	85	30.14	9.00	0.09	0.18	−0.11	−0.04	−0.10	−0.06	—

For every variable, mean and SD as well as Pearson’s correlation index are provided. Columns are numbered according to rows

^*^P < 0.05. **P < 0.01

**Table 6:** Intercorrelations between plasma, urinary oxytocin, and depressive symptom variables at time 2

Variable	*n*	*M*	*SD*	1	2	3	4	5	6	7
1.Plasma oxytocin before interview	82	352.77	121.99	—						
2.Plasma oxytocin after interview	82	340.72	132.61	0.72**	—					
3.Oxytocin in urine	83	193.84	152.22	−0.14	−0.06	—				
4.days since last menstruation	83	15.88	9.67	.264*	0.18	− 0.03	—			
5.PHQ-9	85	6.22	5.56	−0.01	−0.05	−0.20	0.06	—		
6.HAM-D	42	9.31	4.78	−0.06	−0.14	−0.05	−0.06	0.65**	—	
7.Age	85	30.14	9.00	0.10	0.10	−0.18	0.01	−0.20	−0.05	—

For every variable, mean and SD as well as Pearson’s correlation index are provided. Columns are numbered according to rows

^*^P < 0.05. **P < 0.01

### Stability and Change of Plasma and Urinary Oxytocin Through (Successful) Psychotherapy

For the mixed model analysis, only the oxytocin plasma levels before the biographic interview were employed. No significant differences in oxytocin levels were observed between groups (depressed/controls: *F*_(1,159)_ = 0.05; *P* = 0.83). No differences in OT levels at the different time points were observed: *F*_(1,159)_ = 0.41; *P* = 0.52. No two-way interaction between time and group was observed: *F*_(1,159)_ = 0.001; *P* = 0.95 (Fig. 3A). When controlling for possible confounding variables (age, antidepressant medication, hormonal contraception, depressive symptoms (HAM-D)), these findings remained basically the same. None of the possible confounding factors showed any association with plasma oxytocin levels (*data not shown*).

Analyzing the groups in a three-level factorial design (control, remitters, non-remitters), we found again no significant differences (*F*_(2,155)_ = 0.65; *P* = 0.53) and no group × time interaction (*F*_(2,155)_ = 0.02; *P* = 0.98, Table 4).

**Fig. 2:**
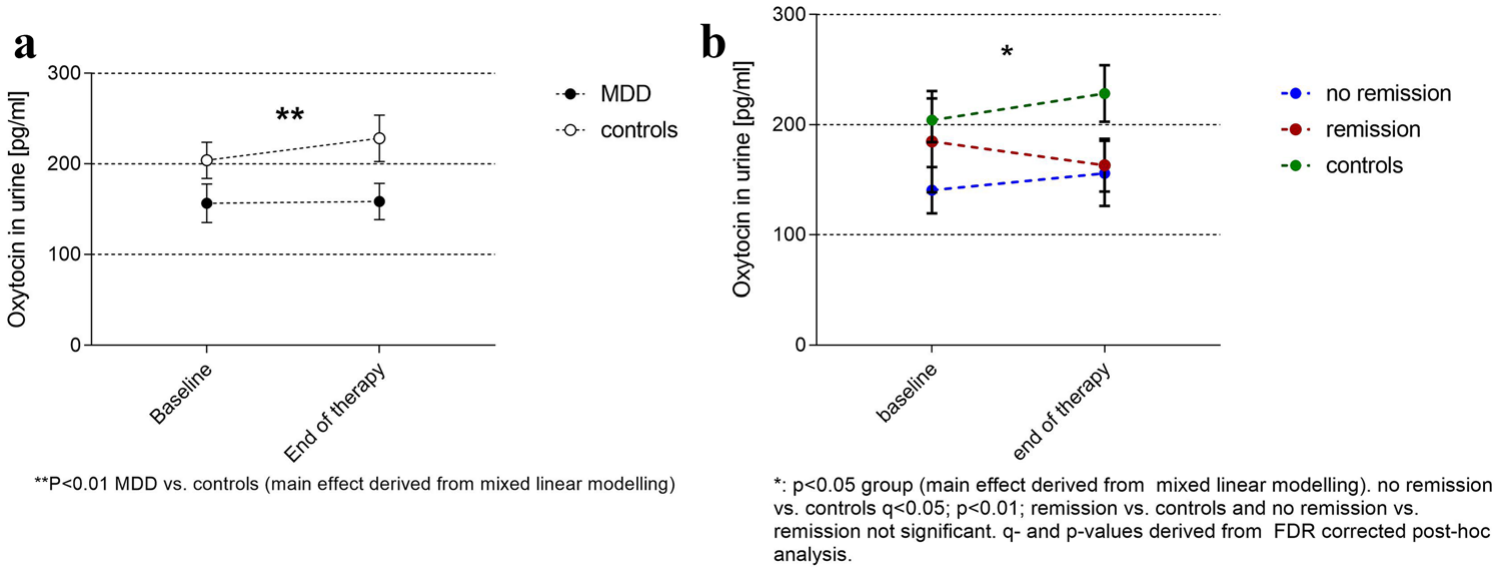
Urinary oxytocin levels. Mean and standard deviation of urinary oxytocin levels at baseline (T1) and end of study (T2) time points in all study participants. As visible in **A**, MDD patients show lower urinary oxytocin levels at both time points without significant changes over time in both groups. Interestingly, this difference is mainly driven by patients not remitting under therapy, as only this group shows significantly lower oxytocin levels than controls (**B**)

Patients had significantly lower urinary oxytocin levels compared with healthy controls (*F*_(1,158)_ = 7.16; *P* = 0.008), while oxytocin levels did not change over time (*F*_(1,158)_ = 0.36; *P* = 0.55) and no time × group interaction occurred (*F*_(1)_ = 1.66; *P* = 0.20, Fig. 2A). None of the possible confounding factors showed any association with urinary oxytocin levels (*data not shown*). Using the three-factorial analysis (s. above), we found that the difference in oxytocin levels between patients and controls was driven by patients not remitting after treatment (*F*_(2;156)_ = 3.78; *P* = 0.025; Fig. 2B).

### Stability and Change of OXTR DNA Methylation Patterns Through (Successful) Psychotherapy

**Fig. 3:**
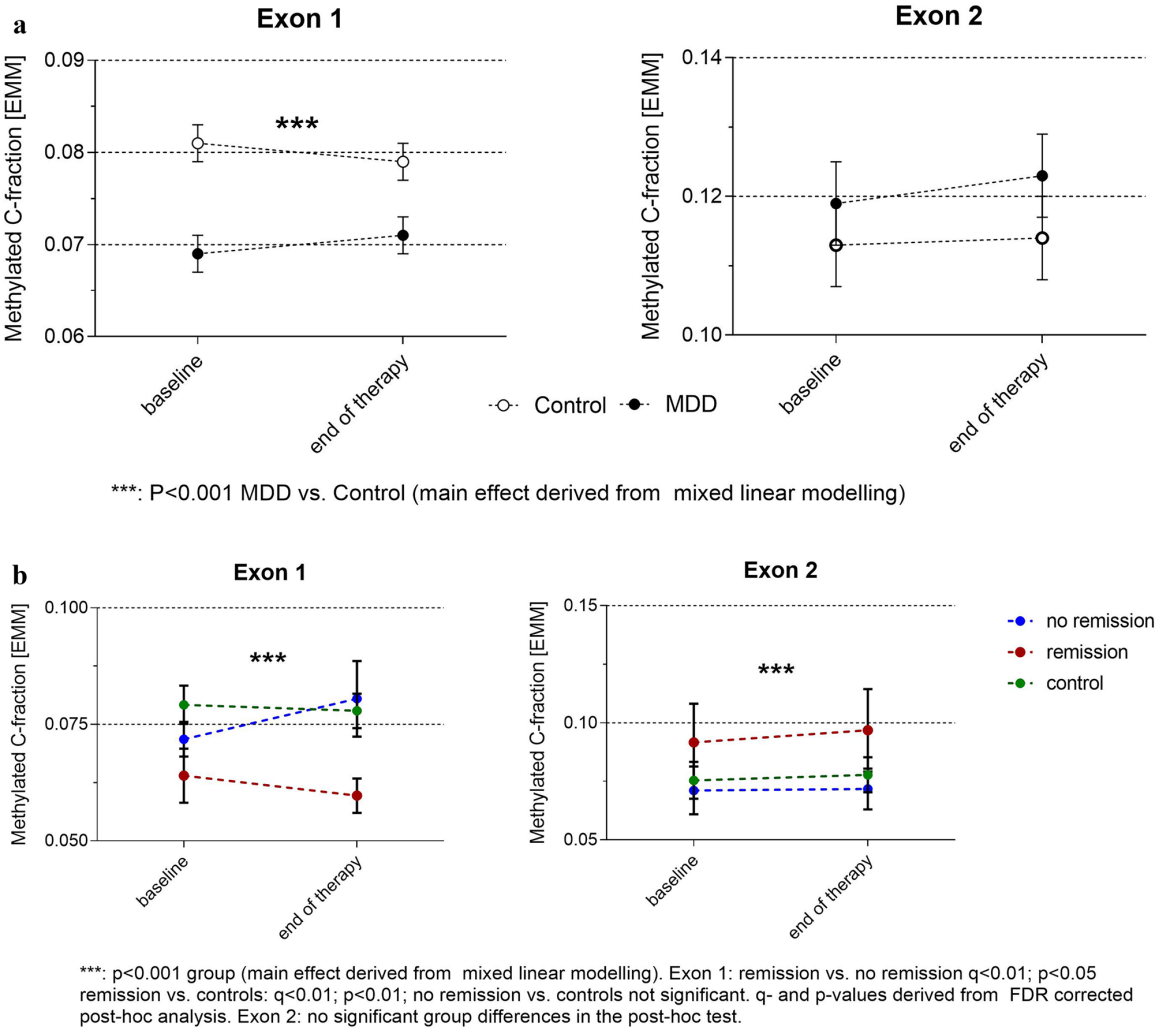
OXTR methylation over time. Methylation of OXTR exons 1 and 2 in the different groups at both time points. Methylation values are provided as estimated marginal means and standard errors derived from the mixed linear modelling (see “Materials and Methods” and “Results” sections). **A** In the group comparison analyzing MDD patients vs. controls, OXTR exon 1 methylation is significantly lower in MDD patients while OXTR methylation levels are stable over time. **B** Comparing three groups (controls, MDD patients remitting under therapy, and MDD patients not remitting under therapy), remitters show lowest OXTR methylation in exon 1 and highest OXTR methylation in exon 2. No significant time point × group interaction occurred. Further details are summarized in the “Results” section

In the mixed linear model including patients and controls at both time points (model 1), we found neither a main effect of time (*F*_(1;6302)_ = 0.05; *P* = 0.821) nor an interaction between group and time (*F*_(1;6302)_ = 0.74; *P* = 0.389). However, we found significant main effects of group (*F*_(1;6302)_ = 12.85; *P* < 0.001) and individual CpG position (*F*_(42;6302)_ = 162.47; *P* < 0.001) as well as a significant CpG × group interaction (*F*_(42;6302)_ = 1.64; *P* = 0.006). Therefore, we repeated the analysis for exons 1 and 2 separately. The main effect of group was only present at exon 1 (Fig. 3A).

We calculated a second model (model 2) with *OXTR* exon 1 methylation at the end of therapy as dependent variable and CpG and group as fixed factors, group × CpG interaction and baseline methylation as covariate. Again, group had a significant effect on *OXTR* exon 1 methylation at T2 (*F*_(1;2459)_ = 8.43; *P* = 0.004) independently of baseline methylation (*F*_(1;2459)_ = 15.50; *P* < 0.001) and CpG position (*F*_(35;2459)_ = 38.81; *P* < 0.001). No CpG × group interaction occurred (*F*_(35;2459)_ = 0.93; *P* = 0.587). The same analysis for exon 2 methylation revealed no effect of group (*data not shown*).

Analyzing group as three-level factor (see above, model 3), we found a significant effect of this variable on *OXTR* DNA methylation (effects of fixed factors are given in Table 7). As estimated marginal means showed only modest differences between groups and a significant CpG × outcome interaction occurred, we repeated the analysis for exon 1 and exon 2 separately. As visible in Fig. 3B, the differences between groups are opposite for both regions: in exon 1, patients remitting under psychotherapy had lower methylation levels at both time points, while patients not remitting under therapy showed comparable methylation levels as controls. In exon 2, patients remitting under therapy had higher methylation levels than both other groups, while patients not remitting under therapy had methylation levels comparable to controls.

**Table 7:** OXTR DNA methylation over time depending on therapy outcome

	Whole fragment		Exon 1		Exon 2	
	*F*_(42.6300)_	*P*	*F*_(35.5258)_	*P*	*F*_(6.1040)_	*P*
Intercept	5869.25	< 0.001	4545.41	< 0.001	1353.56	< 0.001
CpG position	146.07	< 0.001	131.78	< 0.001	181.72	< 0.001
Time point	0.18	0.67	0.02	0.89	0.32	0.57
Group (three levels)	7.77	< 0.001	19.85	< 0.001	7.16	0.001
CpG * time point	0.67	0.951	0.84	0.74	0.23	0.97
CpG * group	1.79	< 0.001	1.23	0.09	1.62	0.08
Time point * group	0.38	0.68	0.71	0.49	0.45	0.64
*N* = 84						

**Fig. 4:**
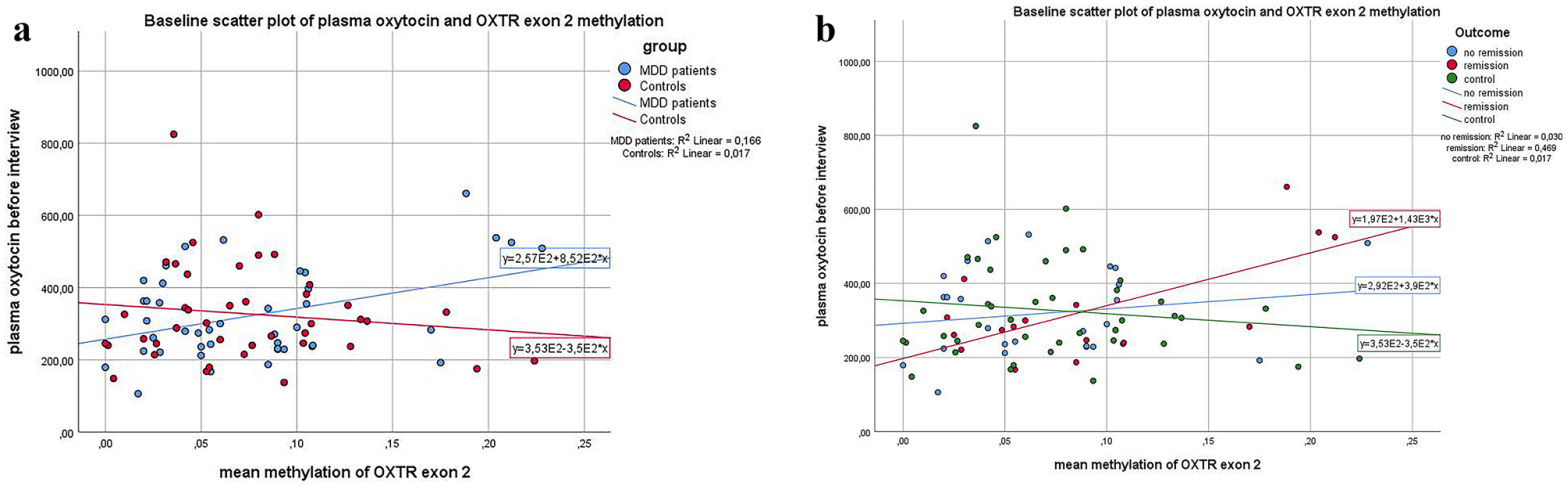
Association between oxytocin plasma levels and OXTR exon 2 methylation. Methylation of OXTR exon 2 and plasma oxytocin levels were positively associated at baseline in MDD patients only (**A**). This positive association was driven by patients later remitting under therapy, while no differences in the distribution were found between controls and patients not remitting under therapy (**B**)

Analyses were carried out for the whole OXTR fragment and exons 1 and 2 separately. Three-level group variable includes controls, patients remitting under treatment, and patients not remitting. *P*-values of the post hoc tests for group are provided in Fig. 4

### Longitudinal Associations Between OXTR DNA Methylation and Urinary and Plasma Oxytocin

We found no correlations between plasma or urinal oxytocin and *OXTR* DNA methylation in the whole study sample. However, when introducing group (patients vs. controls) as possible moderator of the association, we found a significant *OXTR* exon 2 methylation × group interaction on baseline plasma oxytocin levels indicating that an association only existed in the patients group (Table 8 and Fig. 4A). Again, we repeated the analysis to check for a possible difference between patients responding and those not responding to treatment. We found that the association was mainly driven by patients with remission of symptoms (Table 8 and Fig. 4B).

**Table 8:** Association of oxytocin plasma levels and OXTR exon 2 methylation at baseline

*Parameter*	*Estimate*	*SE*	*95% CI*		*P*
			*LL*	*UL*	
**(a) Two-level grouping variable (patients vs. controls)**					
Constant	353.15	34.80	283.89	422.41	< 0.001
Group	−96.09	47.14	−189.91	−2.27	0.05
Mean exon 2 methylation	−350.18	386.23	−1118.80	418.43	0.37
Group * methylation	1202.63	507.96	191.75	2213.51	0.02
**(b) Three-level grouping variable (controls, remitters, non-remitters)**					
Constant	353.15	34.90	283.65	422.65	< 0.001
Group = non-remitters	−60.97	54.73	−169.94	48.01	0.27
Group = remitters	−155.80	64.95	−285.14	−26.47	0.02
Group = controls	0^a^				
Mean exon 2 methylation	−350.18	387.32	−1121.44	421.07	0.37
Group = non-remitters * methylation	740.34	617.68	−489.61	1970.29	0.02
Group = remitters * methylation	1777.02	624.60	533.29	3020.75	0.006
Group = controls * methylation	0^a^				

^a^Redundant, set to zero

We found no further significant associations between plasma or urinal oxytocin and any other methylation measure (*data not shown*).

## Discussion

In a longitudinal, quasi-experimental design, we studied the stability and change of oxytocin-related markers and their associations with outcome to inpatient psychotherapy treatment. To the best of our knowledge, this is the first study that investigated possible associations between plasma oxytocin, urinary oxytocin, and *OXTR* DNA methylation patterns with clinical depression.

Plasma oxytocin levels were not associated with depression in our sample. Mean plasma oxytocin levels did not change over time in controls or after psychotherapy in patients. Plasma oxytocin was also not related to *OXTR* DNA methylation or urinary plasma. Embedding previous conflicting findings on plasma oxytocin levels and depression, our results indicate that the role of plasma oxytocin in clinical depression is imprecise and negligible, at least with current measurement techniques.

Urinary oxytocin levels, in contrast, were significantly lower in depressed patients than healthy controls. Differentiating remitting from non-remitting patients, differences between non-remitting patients versus healthy controls were significant, while the group of remitters was in between those two. Taking subgroups of depression into account, lower urinary oxytocin levels could reflect a chronic course or difficult to treat form of depression. Inpatient psychotherapy focuses mainly on environmental factors that lead and contribute to depressive symptoms. Taking the concept of “endogenous depression” into consideration, urinary oxytocin levels might be a marker of a biologically grounded kind of depression that would need different therapeutic interventions like medication or stimulation therapies to achieve remission. Biochemically, the lowered urinary oxytocin in depressed patients could be explained either by one or more urine metabolites interfering with the antigen–antibody binding in the immunoassay or by an increased proteolytic degradation of oxytocin in depressed versus healthy persons. Oxytocin fragmentation might occur at different steps within the kidney and urinary tract including secretion or shedding processes. So, one could speculate about specific changes in the renal physiology in certain depressed patients. However, further studies are required to validate a link between depression and kidney function. Nonetheless, our findings, if replicated, suggest that urinary oxytocin is a promising marker of clinical depression and potentially of psychotherapeutic outcome.

Further, we found significant lower exon 1 *OTXR* methylation in depressed patients, before and after treatment. This finding validated previously reported findings (Reiner et al. 2015) and expanded knowledge of the stability of *OTXR* methylation patterns. Lower methylation at CpG sites is normally associated with higher transcriptional activity of the corresponding gene and often with higher protein expression at the translation level. Transferred to the results of our study, one may expect increased oxytocin receptor expression in brain areas that are implicated in depression. Interestingly, at least at the mRNA level, this has recently been reported (Lee et al. 2018). In a post-mortem study of persons suffering from major depression, the expression of mRNA for the oxytocin receptor was significantly increased in the dorsolateral prefrontal cortex (Lee et al. 2018). It remains to be shown whether depression is also associated with a higher expression rate of the oxytocin receptor.

Taking treatment response subgroups from our study (remitters/non-remitters) into account, depressed patients remitting though psychotherapy emerged as different while non-remitters and controls did not differ from each other. These treatment subgroups were small and results are in need of replication. However, if replicated, these findings may have implications for understanding the causes of treatment resistance and for treatment planning and prognosis. *OTXR* methylation patterns remain relatively stable and preliminary interpretation allows differentiating treatment responders from non-responders. That is clinically highly relevant and might tap into important insights on psychotherapy efficacy factors in (subtypes of) depression that are based on methylation patterns. Consequently, these results need to be replicated in larger samples and individualized treatment models need to be established for (identifiable) non-remitters-to-be.

Our results reveal that plasma oxytocin, urinary oxytocin, and *OXTR* DNA methylation patterns are intrapersonally relatively stable. While there is some evidence of fluctuations in plasma oxytocin after single psychotherapy sessions (Zilcha-Mano et al. 2018) or a social stressor, in our study, *OXTR-*related factors were seemingly unaffected by long-term interventions such as an 8-week inpatient psychotherapeutic treatment. However, the inpatient psychotherapy led to an overall decrease of depressive symptoms and changes in psychological factors such as attachment security (Reiner et al. 2016).

In conclusion, we found in our longitudinal study the following: (a) plasma oxytocin to be unrelated with depression, (b) lower urinary oxytocin levels in depressed patients, (c) lower exon 1 *OXTR* DNA methylation in depressed patients, and (d) plasma, urinary oxytocin, and *OXTR*-related factors to be statistically unrelated with each other. Remitting patients differed from non-remitting patients regarding urinary oxytocin and *OXTR* DNA methylation, indicating oxytocin-related subgroups of depression.

The findings of this study have to be seen in light of some limitations: First, our study included only females. While this strategy helped to increase the homogeneity of the sample, male-specific, oxytocin-related associations of depression have been ignored. Further studies with male populations are mandatory to fully understand the role of oxytocin in depression as well as possible gender differences or gender-specific associations. Also, the study is not a randomized controlled study, but a quasi-experimental matched control group design with female subjects only. While treatment and control group were selected and recruited carefully and diligently, the sample size is relatively small. Our findings need replication in larger and, as stated above, particularly male samples. Further, plasma oxytocin levels have to be interpreted with caution as it is unknown whether peripheral OT measures reflect central oxytocin release and are reliable indicators of OT-related brain processes (Dal Monte et al. 2014).

## Supplementary Information

Below is the link to the electronic supplementary material.

Supplementary file1 (DOCX 15 KB)

## Data Availability

The datasets used and/or analyzed during the current study are available from the corresponding author on reasonable request.
