# Supplementary material for: OXTR-Related Markers in Clinical Depression: a Longitudinal Case–Control Psychotherapy Study
Source: J Mol Neurosci. 2021 Nov 25;72(4):695–707. doi: 10.1007/s12031-021-01930-7 (PMC8986708; doi:10.1007/s12031-021-01930-7)
Supplement: Supplementary file 1 — Supplementary file1 (DOCX 15 KB) [file 12031_2021_1930_MOESM1_ESM.docx]

Supplementary Table 1: Primers and positions of OXTR fragments

| **Fragment** | **PCR** | **Primer** | **Sequence** | **Tm (°C)** | **Position**  **in relation to**  **Exon 1 (bp)** | **Product size (bp)** | **Location on Chromosome 3**  **(alternate assembly HuRef)** |
| --- | --- | --- | --- | --- | --- | --- | --- |
| A | 1A | OXTR_A_F1_80 | TTATTGTAAAATAAATTTATTTGTTAAGG | 56 | -21 to + 405 | 427 | >gi\|157731950\|ref\|AC_000135.1\|:8744960-8745386 |
|  |  | OXTR_A_R2_83 | CCCCTACTTAACACCCAACTACCT | 65 |  |  |  |
|  |  |  |  |  |  |  |  |
|  | 2A | OXTR_A_F2_82 | AATTTATTTGTTAAGGTTTTGGGA | 55 | -8 to +400 | 409 | >gi\|157731950\|ref\|AC_000135.1\|:8744965-8745373 |
|  |  | OXTR_A_R1_81 | ACTTAACACCCAACTACCTACACC | 64 |  |  |  |
|  |  |  |  |  |  |  |  |
| B | 1B | OXTR_B_F1_88 | GGTGTAGGTAGTTGGGTGTTAAG | 63 | +376 to +694 | 318 | >gi\|157731950\|ref\|AC_000135.1\|:8744671-8744988 |
|  |  | OXTR_B_R2_91 | CTACTAAAAAAACCCCTACCTCAA | 60 |  |  |  |
|  |  |  |  |  |  |  |  |
|  | 2B | OXTR_B_F2_90 | GTAGGTAGTTGGGTGTTAAGTAGGG | 66 | +379 to +682 | 303 | >gi\|157731950\|ref\|AC_000135.1\|:8744683-8744985 |
|  |  | OXTR_B_R1_89 | CCCCTACCTCAAAACCCAAAATAC | 64 |  |  |  |
|  |  |  |  |  |  |  |  |
| C | 1C | OXTR_C_F1_84 | GGGGAAGGATTAGAGTTGGGGTTT | 65 | +780 to +1205 | 425 | >gi\|157731950\|ref\|AC_000135.1\|:8744160-8744584 |
|  |  | OXTR_C_R2_87 | AAAAACCCAACTCATCTAAAACA | 56 |  |  |  |
|  |  |  |  |  |  |  |  |
|  | 2C | OXTR_C_F2_86 | TTAGAGTTGGGGTTTTTTAGGTAA | 58 | +789 to +1198 | 409 | >gi\|157731950\|ref\|AC_000135.1\|:8744167-8744575 |
|  |  | OXTR_C_R1_85 | CAACTCATCTAAAACAACAAAACACAACC | 65 |  |  |  |
|  |  |  |  |  |  |  |  |
